# Cognitive behavioural therapy plus Kiesler Circle Training (CBT+) versus CBT only for patients with interpersonal problems: study protocol for a randomised controlled feasibility trial

**DOI:** 10.1136/bmjopen-2024-098466

**Published:** 2025-02-11

**Authors:** Karen Ollrogge, Daniel Schulze, Philipp Sterzer, Stephan Köhler, Eva Friedel, Nina Romanczuk-Seiferth, Anna-Lena Siegerist, Eva-Lotta Brakemeier, Anne Guhn

**Affiliations:** 1 Psychiatry and Neurosciences, Charité - Universitätsmedizin Berlin, Berlin, Germany; 2 Institute of Biometry and Clinical Epidemiology, Charité - Universitätsmedizin Berlin, Berlin, Germany; 3 Psychiatry and Psychotherapy, University Psychiatric Clinics Basel, Basel, Switzerland; 4 Department of Psychiatry, Psychotherapy and Psychosomatics, St Joseph Hospital Berlin Weissensee, Berlin, Germany; 5 Psychology, MSB Medical School Berlin GmbH, Berlin, Germany; 6 Department of Clinical Psychology and Psychotherapy, University of Greifswald, Greifswald, Germany

**Keywords:** Clinical Protocols, Anxiety disorders, Depression & mood disorders, Psychosocial Intervention, Randomised Controlled Trial

## Abstract

**Introduction:**

Interpersonal problems are a transdiagnostic risk factor for the development and maintenance of various psychiatric disorders, including anxiety and depression. Interventions that address these interpersonal challenges could therefore play a crucial role in enhancing mental health. This study aims to evaluate the efficacy and feasibility of a transdiagnostic group intervention, the Kiesler Circle Training (KCT), in improving interpersonal skills.

**Methods and analysis:**

In a prospective randomised controlled trial (RCT), 156 outpatients with a primary diagnosis of either depression or anxiety disorder according to DSM-5, and significant interpersonal problems, will be investigated. All patients will receive individual, state-of-the-art cognitive behavioural therapy (CBT) during the study. They will be randomly assigned to one of two conditions: the experimental group will receive the KCT in group sessions, in addition to individual CBT, while the control group will receive only individual CBT. The KCT intervention consists of 1 introductory individual session and 12 weekly group sessions, each lasting 100 min, with groups of up to 10 patients. KCT includes five sequential modules: the interpersonal circle, nonverbal communication, verbal communication, conflict resolution and empathy training. It is hypothesised that the experimental group will show (a) greater reduction in interpersonal problems from pre-assessment to post-assessment and (b) greater symptomatic improvement regarding the primary diagnosis. Child maltreatment is expected to moderate the trajectory of interpersonal problems. This study aims to provide evidence for the feasibility of KCT as a modular transdiagnostic add-on approach for patients with interpersonal difficulties.

**Ethics and dissemination:**

This study obtained approval from the ethics committees at the Charité Berlin, the Medical School of Berlin and the University of Greifswald. All results will be disseminated through peer-reviewed articles in scientific journals and contributions to national and international conferences.

**Trial registration number:**

DRKS00032467, NCT06170801 (see Supplementary Material).

**STRENGTHS AND LIMITATIONS OF THIS STUDY**

- The Kiesler Circle Training (KCT) is an innovative group therapy approach that addresses interpersonal problems as a transdiagnostic process.
- The study will provide point estimates for the efficacy of KCT on standardised symptom measures, focusing on primary (interpersonal problems by self-report referring to the transdiagnostic approach) and secondary endpoints (clinical measures by blinded observers referring to the categorical approach), including side effects.
- Following the transdiagnostic approach, comorbid mental disorders, including some personality disorders, are included in the study, allowing for a broader and more representative patient sample.
- The study is limited by the fact that the intervention group receives a higher frequency of therapy sessions than the control group.
- The sample size is determined to detect a meaningful treatment effect but may be insufficient to identify potential moderators of the treatment effect.

## Introduction

 In recent decades, there has been a shift in the conceptualisation of mental disorders. Moving away from a categorical approach centred on discrete diagnostic groups, the field has adopted a transdiagnostic perspective advocating for a dimensional understanding of psychopathology.^1^ The categorical approach, notably used by the current Diagnostic and Statistical Manual of Mental Disorders (DSM-5) and the International Classification of Diseases (ICD-11), proposes a group of symptoms to define specific disease entities. In this framework, symptoms are seen as manifestations of an underlying common cause, which requires disorder-specific interventions.^2^ However, the categorical approach fails to account for the high comorbidity rates observed across disorders^3^ and does not explain why disorder-specific treatments often lead to improvements in symptoms that were not the primary focus of the intervention.^4 5^

In contrast, the transdiagnostic approach argues that many disorders share key etiological processes that transcend traditional diagnostic boundaries, with the interplay of these processes playing a causal role in the emergence and maintenance of psychopathology across diagnostic categories. The transdiagnostic approach calls for innovative and effective treatment strategies^6 7^ that target the various domains of transdiagnostic processes, including attention, memory, reasoning, thought and behaviour.^8^

### Interpersonal problems

The present study focuses on interpersonal functioning as a key transdiagnostic process within the behavioural domain. Interpersonal problems are defined as difficulties that occur persistently and repetitively in relationships with others and cause distress.^9^ Interpersonal problems are by definition a hallmark of personality disorders, but they also manifest across a range of other psychiatric conditions. Patients with psychiatric diagnoses experience significantly more interpersonal difficulties than healthy reference groups^10^ and report higher levels of general distress in social interactions.^11^ These difficulties are a small but robust negative predictor of psychotherapy outcomes.^9^ As such, addressing interpersonal issues within therapy is likely to enhance overall therapeutic effectiveness.

Deficits in interpersonal functioning have been linked to early maladaptive interactions with significant others. Child maltreatment, especially emotional abuse and neglect,^12^ is a well-established predictor of depression and anxiety.^13 14^ It is closely associated with heightened interpersonal difficulties,^15 16^ likely due to its impact on the ability to form and maintain healthy relationships in adulthood.^17^ According to a recent meta-analysis, child maltreatment is associated with lower global social functioning (r = −0.11 to −0.20) and poorer interpersonal relations (r = −0.18 to −0.33).^18^ While the impact of child maltreatment on the course of treatment remains inconclusive,^19 20^ there is evidence that psychotherapeutic approaches that consider the biographical background are particularly effective. Patients with distinct patterns of emotional neglect and abuse demonstrated better treatment outcomes with the Cognitive Behavioral System of Psychotherapy (CBASP) compared with non-specific supportive psychotherapy.^21^ These findings emphasise the importance of psychotherapeutic interventions addressing the interpersonal impact of child maltreatment, such as those applied in the Kiesler Circle Training (KCT).

### Improving interpersonal skills: the Kiesler Circle Training (KCT)

**Figure 1:**
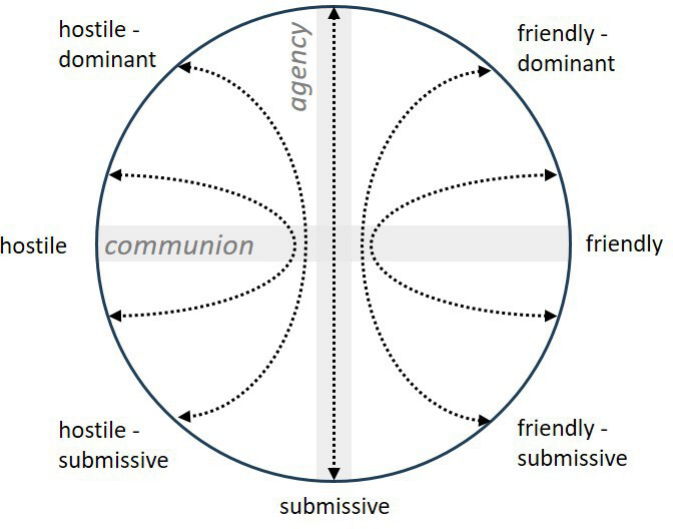
Kiesler’s circumplex model. The x axis depicts communion; y axis depicts agency. Arrows illustrate interpersonal action tendencies.

The KCT is based on an empirically derived structural model used to organise interpersonal functioning within the Contemporary Integrative Interpersonal Theory (CIIT).^22^ This model arranges the variety of interpersonal behaviours in terms of a circular continuum allocated on two orthogonal axes: the vertical axis addresses interpersonal control (agency), which ranges from dominant to submissive behaviour, whereas the horizontal axis addresses interpersonal communion, which ranges from friendliness to hostility (figure 1). The circumplex model allows illustrating complementary action tendencies to anticipate others’ reactions and to guide one’s own behaviour to achieve interpersonal goals. Patients with anxiety and depression are often associated with a rigid pattern of socially avoidant interpersonal behaviour,^23 24^ referring to low agentic and low communal behaviours. KCT is designed for increasing the awareness of interpersonal action tendencies and for improving interpersonal behavioural flexibility. It is based on the individual biographical background, the so-called significant-other history, and targets corrective interpersonal experiences against former interpersonal experiences of child maltreatment. KCT originated from CBASP,^25^ but was adopted and manualised for the group setting.^26^ In a phase I study conducted in an inpatient CBASP setting, patients with the primary diagnosis of persistent depressive disorder along with high rates of interpersonal problems and child maltreatment demonstrated reduced interpersonal problems after 12 weeks of treatment including weekly KCT group sessions.^27^ The reduction of interpersonal problems was moreover significantly associated with the reduction of depression severity (r=0.57). However, the homogeneous patient group, the inpatient setting and the absence of a control group limit the interpretation regarding the efficacy and feasibility of KCT as transdiagnostic group intervention.

### Aims and hypotheses

The present study aims to investigate the efficacy and feasibility of a transdiagnostic GRroup intervention for improving InterPersonal Skills (study acronym: GRIPS), the KCT, by comparing individual, state-of-the-art cognitive behavioural therapy (CBT) augmented by KCT group sessions with individual CBT only in patients with a categorical diagnosis of either depression or anxiety and significant interpersonal problems. Interpersonal distress represents the primary outcome. Considering the widespread prevalence of interpersonal problems across various categorical diagnoses, including mood disorders, anxiety and phobic fears, psychosis, substance use disorders, eating disorders and personality disorders,^10 11 28^ the reduction of interpersonal distress is supposed to accompany symptomatic change of the respective categorical diagnosis. The main hypotheses are:

1. Compared with CBT alone, the combination of CBT and KCT (CBT+) is expected to result in a greater reduction in interpersonal problems from pre-assessment to post-assessment.
2. The reduction in interpersonal problems will be associated with greater symptomatic improvement in the primary diagnosis, with patients in the CBT+ condition demonstrating greater symptom reduction compared with those receiving CBT alone.

The study will also examine secondary hypotheses related to moderating and mediating effects on interpersonal functioning. Specifically, we will test the hypothesis that the impact of KCT on reducing interpersonal problems is moderated by experiences of child maltreatment, with individuals reporting higher levels of maltreatment benefiting more from the intervention. Additionally, we hypothesise that the quantity and quality of daily social interactions mediate these improvements.

## Methods and analysis

### Study design

This prospective randomised controlled phase II trial (RCT) compares two active conditions: CBT in individual sessions augmented by 12 weekly KCT group sessions (experimental group, CBT+) versus CBT in individual sessions only (control group, CBT). Individual patients will be randomised 1:1 to each condition, with randomisation conducted separately at each study centre and stratified by primary diagnosis (depression or anxiety) to ensure balanced group distribution. The allocation sequence will be generated using computer-generated random numbers. The study will be conducted at the Centre for Psychological Psychotherapy (ZPP) at the University of Greifswald, Germany, and the Institute for Integrative Psychotherapy Training (IPB) at the Medical School Berlin, Germany. Both sites will conduct CBT+ and CBT only in an outpatient setting. After study inclusion, patients will be assessed at baseline (T1, weeks 1–2), mid-treatment (T2, week 8), post-treatment (T3, week 14) and follow-up (T4, 3 months later).

### Study population and recruitment

156 patients with a categorical diagnosis of either a depressive or an anxiety disorder according to DSM-5 and significant interpersonal problems (one SD above the German norm according to Thomas *et al*^29^) will be recruited. Patients eligible for the trial must comply with the following inclusion criteria at randomisation:

- Age: 18–70 years.
- Sufficient knowledge of the German language.
- A primary diagnosis of either depressive disorder (major or persistent depressive disorder) or anxiety disorder (specific phobia, social anxiety disorder, panic disorder, agoraphobia, generalised anxiety disorder) according to DSM-5.
- Interpersonal distress above average (IIP-32 >1.81).
- Ongoing individual CBT at study entry.
- Signed informed consent after verbal and written explanation by the study coordinator.

Key exclusion criteria are the following:

- Acute suicidality.
- Active substance abuse.
- A previously known diagnosis of autism.
- A diagnosis of borderline, antisocial, schizoid or schizotypic personality disorder.
- Inability to participate in the weekly Kiesler Circle Training group therapy.
- Any kind of additional group treatment (including self-help groups) besides individual CBT during the entire study period.

Study inclusion is possible at any point during the course of ongoing individual CBT. To ensure sufficient patient recruitment in the planned period of 20 months, the study will be regularly announced in person and by flyers and newsletters.

To lower dropouts, patients randomised to the control group will be offered to join the KCT group therapy after completion of the FU assessment (T4). These patients will be asked to answer the evaluation forms for the KCT modules, which will increase the amount of feedback that contribute to the improvement of the KCT manual for a future study. Moreover, all patients will get an expense allowance for taking part in the assessments at T2 and T4.

#### Sample size

**Figure 2:**
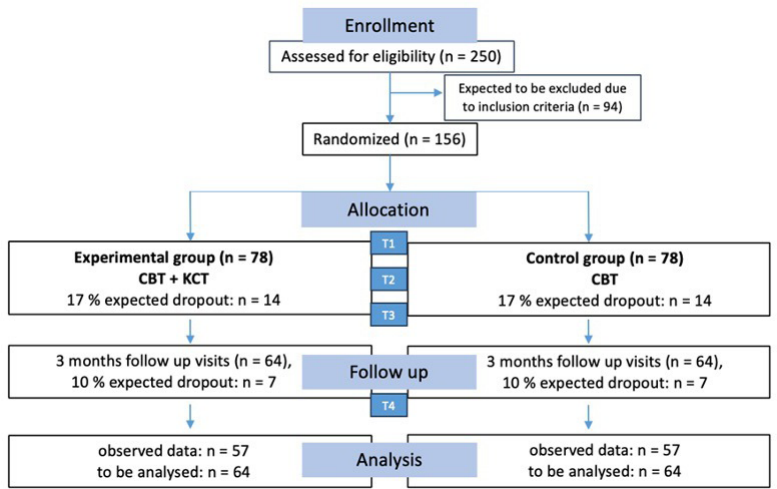
Trial design. CBT, cognitive behavioural therapy; KCT, Kiesler Circle Training; T1, baseline; T2, mid-assessment; T3, post-assessment; T4, follow-up.

The sample size calculation is based on Holgersen *et al*,^30^ who argue that a clinical significance can only be assumed with an effect size of Cohen’s d=0.5, considering the costs of implementing an additional group therapy. Our sample size calculation relies on a two-tailed two-sample t-test, while a baseline-adjusted analysis of covariance (ANCOVA) will be used for the primary efficacy analysis. This results in a higher power compared with the two-sided t-test, which ignores the influence of different baseline values. This strategy for calculating the sample size is a conservative procedure. With a two-sided t-test with equal variance, the power analysis resulted in a group sample size of 64. This sample size achieves a power of 80% for rejecting the null hypothesis of equal means at a significance level (alpha) of 0.05. Considering approximately 17% dropouts during the treatment phase based on our phase I study,^26^ the total number of patients to be recruited is 156 (78 per group). Consequently, approximately 250 patients will undergo eligibility screening using diagnostic tools (see figure 2).

#### Outcome measures

Change in interpersonal distress by self-report from baseline (T1) to post-assessment (T3) represents the primary outcome (Hypothesis 1). Interpersonal distress will be assessed by the IIP-32,^29^ a short version of the original IIP-D.^31^ Individuals have to rate the extent to which a number of behaviours, thoughts and feelings in social interactions pose difficulties for them on 32 Likert-scaled items that range from 0 (not at all) to 4 (absolutely). The mean score of all items indicates the general level of interpersonal distress; higher values represent more severe interpersonal problems. The IIP-32 shows a high validity^29^ and a satisfactory sensitivity to change.^32^

Symptom severity changes with regard to the primary diagnosis (Hypothesis 2) from T1 to T3 will be assessed by independent and trained raters who are blinded for treatment assignment. Patients are advised not to reveal any information that might indicate their group assignment during the trial visits. In case the group assignment is revealed, a back-up rater will conduct the pending trial visits. For patients with a primary anxiety disorder, the 14-item version of the Hamilton Anxiety Rating Scale (HAM-A) will be investigated. For patients with a primary depressive disorder, the 24-item version of the Hamilton Rating Scale for Depression (HAM-D) will be used. HAM-A and HAM-D are semistructured interviews with good psychometric qualities^33 34^ that measure the severity of all symptom domains of anxiety and depression described by the DSM over the last 7 days. Change sum scores from pre-assessment (T1) to post-assessment (T3) will be standardised into z-values so that HAM-A and HAM-D scores can be combined for the analysis. The reliable change index (RCI) will be calculated to assess whether difference scores can be considered clinically meaningful.

Child maltreatment will be assessed as moderator of change using the Childhood Trauma Questionnaire.^35^ Ecological Momentary Assessment (EMA) will be used to identify mediators of change in interpersonal functioning and symptom severity. EMA involves collecting real-time data in the natural environment of participants via smartphones, allowing to capture the dynamics of interpersonal behaviour and emotional states as they unfold.^36^ In this study, data will be collected four times per day at random intervals. At each assessment, participants will report on core characteristics of their behaviour (quality of social interactions) in their most recent social interaction, the number of social interactions since the last assessment (quantity of social interactions), and their affective state. Participants can decide whether to consent to smartphone data collection and join the additional GRIPS-EMA study. This decision will not affect their continued participation in the GRIPS study.

A comprehensive overview of the frequency and scope of all trial visits is depicted in table 1. Table 2 summarises primary and secondary outcomes with its corresponding measures. Exploratory outcomes are summarised in a supplementary table (see onlinesupplemental tables 1^ 2^).

**Table 1:** Frequency and scope of trial visits

	Screening	Baseline	Intervention						FU
Trial visits	T0	T1			T2			T3	T4
Week	0	1–2	4	6	8	10	12	14	26
Informed consent	X								
Inclusion/exclusion criteria	X								
SCID-5-CV	X								
SCID-5-PD	X								
Demographic information (OR)	X								
Randomisation*	X								
Primary outcome									
IIP-32 (SR)	X	X			X			X	X
Secondary outcome									
Symptom severity (OR)—depression: HAM-D—anxiety: HAM-A		X			X			X	X
Moderator of change									
CTQ (SR)		X							
Mediator of change									
EMA		X			X			X	
Exploratory outcome									
Symptom severity (SR) depression: BDI-V, anxiety: BCQ and ACQ		X			X			X	X
IMI-R (OR)		X						X	X
IIM (SR)		X						X	X
SEDI (SR), PHQ (SR)		X						X	X
WHOQoL-BREF (SR)		X						X	X
PID5BF+ (SR)		X						X	X
LPFS-BF (SR)		X						X	X
CAMSQ (SR)		X						X	
Adverse events and side effects									
Medication†		X						X	X
Side effects (INEP)								X	
NUGE-24 (CBT+KCT only)					X			X	X

* Randomisation will be performed by the CTO Charité

† There are no specific guidelines regarding medication, as prescriptions are made outside of psychotherapy sessions; however, any changes to medication are documented

ACAquestionnaire on panic-related Anxieties, Cognitions and Avoidance (subscales Body Sensations Questionnaire (BSQ) and the Agoraphobic Cognitions Questionnaire (ACQ))BDI-VBeck Depression Inventory, simplifiedCAMSQCertainty About Mental States QuestionnaireCTQChildhood Trauma QuestionnaireEMAEcological Momentary AssessmentHAM-AHamilton Anxiety Rating ScaleHAM-DHamilton Depression Rating ScaleIIMInventar zur Erfassung Interpersoneller MotiveIIP-32Inventory for Interpersonal Problems, short versionIMI-RImpact Message Inventory, revised versionINEPInventory for the Assessment of Negative Effects of PsychotherapyLPFS-BFLevel of Personality Functioning Scale-Brief Form 2NUGE-24Questionnaire for the Assessment of Side Effects and Negative Experiences in Group TherapyORblinded observer-ratingPHQPatient Health Questionnaire (subscales PHQ-9, PHQ-15 and GAD-7)PID5BF+Personality Inventory for DSM-5 Brief Form PlusSAEsserious adverse events monitoringSCID-5Structured Clinical Interview for DSM-5 (CV, clinical version; PD, personality disorders)SEDIshort version of the Multidimensional Emotional Disorder InventorySRself-rating

**Table 2:** Primary and secondary outcomes with its corresponding measures

Outcome measure	Measure description
Primary outcome	
Inventory of Interpersonal Problems (german version)^29^: IIP-32^43^	The IIP-32 is a self-report form for interpersonal problems based on Horowitz's Circumplex Model. It consists of 32 items that assess difficulties in interacting with others on a 5-point Likert scale (0=not at all, 4=very much).
Secondary outcomes	
Hamilton Depression Rating Scale, 24 items version HAM-D^44^	The HAM-D is a semistructured interview, which measures the severity of depressive symptoms experienced in the last week. Blinded and trained study raters will assess the severity of symptoms for each of the 24 items using a scale ranging from 0 to 2, 0–3, or 0–4. The total score ranges from 0 to 75, with higher scores indicating a higher severity of depression.
Hamilton Anxiety Rating Scale HAM-A^34^	The HAM-A is a semistructured interview, which measures the severity of anxiety symptoms experienced in the last week. Blinded and trained study raters will assess the severity of symptoms for each of 14 items using a 5-point scale ranging from 0 to 4. The total score ranges from 0 to 56, with higher scores indicating a higher severity of anxiety.
Other outcome measures (moderation and mediation)	
Childhood Trauma Questionnaire (german version)^35^: CTQ^45^	Child maltreatment is assessed as a main moderator at baseline. The CTQ is a self-report assessment designed to measure experiences of child trauma and abuse on five subscales (emotional abuse, physical abuse, sexual abuse, emotional neglect and physical neglect). Patients rate the frequency of 34 of these experiences on a 5-point scale (1=not at all, 5=very often), and each subscale score has a range from 5 to 25 points. Higher scores indicate a higher severity in child trauma.
Quality and quantity of daily social contacts	Patients answer short questionnaires four times a day, which appear on their smartphone at random times (Electronical Momentary Assessment, EMA). The number of social contacts, the quality of social contacts (interpersonal complementarity), interaction partner, affect and anxiety are assessed.

### Interventions

#### Experimental intervention: KCT

The KCT is a transdiagnostic group therapy developed for patients with interpersonal problems.^26 27^ Within the framework of this study, KCT will be investigated as augmentation of individual state-of-the-art CBT. KCT consists of 12 sessions of 100 min each in a group of up to 10 patients. One individual session of 100 min will be conducted before the beginning of the group with one of the two group therapists, both to establish a working alliance, given the concerns and worries patients with interpersonal problems might have before entering the group, and to develop a so-called individual transference model to anticipate upcoming interpersonal problems. This model assumes that negative experiences with significant others from the past translates into patients’ expectations about what is likely to happen in current social interactions. Therapists help their patients to (a) identify their individual interpersonal problems, (b) connect their past experiences with others to their present interpersonal fears (transference hypotheses) and (c) define individual treatment goals for the group sessions. The group therapy comprises five modules: (a) getting to know the interpersonal circle, (b) nonverbal communication, (c) verbal communication, (d) conflict training, (e) empathy and corrective interpersonal experiences. Group sessions are manualised but designed flexibly in a way that some interventions in each module are mandatory while others are flexible (box 1). All modules comprise both psychoeducative elements and experience-activating methods, such as role-play, to actively acquire and train interpersonal skills.

Box 1 **Overview of the Kiesler Circle Training modules**

**Module 1: getting to know the Kiesler Circle—minimum number of sessions: 2**

This module introduces the Kiesler Circle as a core analytical framework for understanding interaction patterns. Participants learn to recognise their own behavioural patterns and their impact on others.

Mandatory content: the Kiesler Circle is introduced with its two axes (agency and communion) and the concept of interactional tendencies. Interactive elements such as role-plays, where therapists demonstrate different positions on the Kiesler Circle, and an exercise in which participants match cards with adjectives to the corresponding positions on the Kiesler Circle help participants getting familiar with the model.

Optional content: participants create their own Kiesler Circle profile, reflecting on past experiences in significant relationships to identify behaviours that are typical for them. If the group atmosphere becomes tense or participants express anxiety, feedback rules help to address potential transference issues and ensure a supportive environment.

**Module 2: nonverbal communication—minimum number of sessions: 1**

Understanding and interpreting nonverbal communication is essential for developing interpersonal skills. This module equips participants with the tools to ‘read’ unspoken signals accurately, allowing for better adaptation and responses in real-world situations.

Mandatory content: in the discrimination training, patients work in groups to identify how the eight KC positions differ in terms of key attributes of nonverbal communication: gait, posture, gestures, and facial expressions. Based on the concept of interactional action tendencies, the positions are then pantomimed and explained.

Optional content: in the circuit training, patients pantomime specific KC positions, followed by group reflection. The optional facial expression session can focus in more detail on the facial expression (forehead/eyes, mouth, head position) of the various Kiesler Circle positions. The differentiation of the agency and communion axes can be deepened using the exercises Leading and Following and Closeness and Distance. In Leading and Following (control axis), patients pair up, and one person closes their eyes while the other guides them through the room. In Closeness and Distance (affiliation axis), pairs become playfully aware of how much proximity they feel comfortable allowing and how to signal the need for distance when necessary. Both exercises are followed by group reflection.

**Module 3: verbal communication—minimum number of sessions: 2**

This module addresses verbal aspects of social interactions, helping participants to articulate their needs clearly and interpret the verbal cues of others correctly, thus reducing potential misunderstandings.

Mandatory content: in the discrimination training, short role-plays are used, in which both therapists and patients speak short sentences in front of the group to highlight the different KC positions based on voice and volume, typical word choice, and relationship dynamics. The SEW (Situation, Emotion, Wish)-scheme, which serves as a guideline for expressing needs or desires, is introduced and practiced using various examples.

Optional content: the SEW-scheme can be further deepened through role-playing exercises. The Party Game is an exercise where the group collaboratively plans a party in a role-play scenario. Each participant is secretly assigned a specific KC position beforehand, which is then followed by a group reflection on the dynamics observed during the activity.

**Module 4: conflict training—minimum number of sessions: 2**

This module is designed to build on previously learned skills and apply them to real-world conflict situations. Participants learn structured approaches to analysing and resolving conflicts constructively.

Mandatory content: in the anger psychoeducation session, participants explore the challenges of addressing and resolving conflicts due to emotions like disappointment, anger, or helplessness. They learn that avoiding these feelings (hostile or hostile-submissive Kiesler Circle positions) often leads to negative outcomes, while open and direct communication (friendly-dominant) is more beneficial. The session can be introduced either through an open discussion on the nature of anger, focusing on healthy versus unhealthy expressions, or by using a four-field table exercise that highlights the short- and long-term consequences of different anger responses using a fictional scenario. Participants learn about the escalation levels on the Kiesler Circle, which serve as a framework for resolving conflicts. They are encouraged to start at Level 1 (friendly-dominant), move to Level 2 (dominant) if needed, and only use Level 3 (hostile-dominant) in exceptional cases, as it can potentially damage or end relationships. The escalation levels are practiced using concrete examples.

Optional content: in the self-confidence training, patients learn to say ‘no’ by rejecting a request from the therapist in a role-play. The deepening of the escalation levels can be trained using predefined scenarios and role-playing games. A session on ‘saying yes and no’ can be conducted: discussions and role-plays can be used to explore the short and long-term consequences of saying yes and no.

**Module 5: empathy and corrective relationship experiences—minimum number of sessions: 1**

This module helps participants strengthen their capacity for empathy and mutual understanding, thereby enhancing the quality of their relationships and preparing them for the closure of the group process.

Mandatory content: in the Paul and Paula empathy training, therapists role-play a partnership conflict to help patients practice perspective-taking. The final session is dedicated to self-reflection, where participants are encouraged to recall earlier sessions and consider how their fears, impressions, and expectations have evolved throughout the KCT. Additionally, transference hypotheses developed during the individual session prior to the start of the group should be revisited and discussed.

Optional content: in the Warm Back exercise, participants receive positive notes on sheets taped to their backs, which can be discussed afterwards. The Trust Fall, an exercise in which one person gently falls while trusting others to catch them, encourages reflection on group cohesion and belonging. The Interpersonal Discrimination Exercise (IDE) involves comparing current interactions with past significant relationships to correct or enhance experiences. At the end of the group, therapists can conduct an IDE for the entire group by positively discussing their observations about the group as if the members were not present (similar to Reflecting Teams).

#### Control intervention: CBT

Modern, state-of-the-art individual CBT serves as the control intervention. At its core, CBT is based on the interaction between thoughts, emotions and behaviour. Individual CBT for depression and anxiety primarily focuses on *intrapersonal* therapeutic areas, such as identifying and modifying dysfunctional thoughts, promoting behavioural activation, conducting fear exposure, implementing behavioural experiments or teaching relaxation skills. Additionally, it may address *interpersonal* areas, such as social skills training and enhancing social competence. Modern, state-of-the-art CBT also integrates concepts from the so-called ‘third wave’ of therapy, including the exploration of negative schemas, skills acquisition, mindfulness or acceptance-based strategies. In line with the categorical approach, disorder-specific treatment manuals are available for all diagnoses included in the study. However, state-of-the-art CBT does not necessarily entail strict adherence to a manual. Since both study sites are CBT training institutes that ensure supervision at least every 4 hours, it can be reasonably assumed that modern, state-of-the-art CBT is being implemented. Individual therapists will be asked to document the interventions used in each session via a checklist.

#### Adherence

Study therapists at both study sites are graduated psychologists who are at an advanced stage of postgraduate psychotherapy training. Study therapists who guide KCT participated in a full-day workshop in which the KCT modules were trained according to the group manual and challenging interpersonal situations within the group were trained by demonstration and role-play. Before treating study patients with KCT, therapists must meet specific competence criteria based on the CBASP adherence rating regarding the therapeutic relationship. These criteria include collaboration, therapeutic empathy towards the patient, effective listening, appropriate control, tolerance of negative affects, and the use of therapeutic self-disclosure when appropriate. Competence is assessed by evaluating a videotaped individual session in which a patient’s individual transference model is developed. The results of the rating are discussed in an individual supervision session before the start of group therapy, and, if necessary, modifications for behaviour change are identified and developed. After that, biweekly group supervision is mandatory for study therapists to ensure treatment adherence. KCT group sessions will be videotaped for supervision.

### Data management and monitoring

Study data will be entered in pseudonymised form in a study database by authorised and trained members of the study team via electronic case report forms (eCRF). Self-reports are entered automatically into eCRF by patients. Data capture and data management will be performed using the study software SecuTrial (interActive Systems, Berlin) provided by Charité. SecuTrial features remote web-based data capture and real-time monitoring. The software has been designed to meet the requirements of the FDA (21 CFR Part 11) and the guidelines for Good Clinical Practice.

### Statistical analysis

The analyses of the primary and secondary endpoints will be performed by a statistician blinded to treatment allocation. Statistical analysis will be performed according to the intention-to-treat principle and longitudinal dropouts will be handled via multiple imputation. Regarding the first hypothesis, primary analysis will consist of an ANCOVA for the treatment effect (time by group), adjusted for interpersonal distress at baseline, the number of individual sessions between T1 and T3, and treatment site. Regarding the second hypothesis, that is, mediation of the treatment effect in primary diagnosis by reductions in interpersonal distress, longitudinal structural equation models will be applied for disentangling causal effects. Here, measurements taken at T2 will serve as intermediate measurement. To test the moderator hypothesis about child maltreatment, a further ANCOVA will be conducted, including interaction effects. Sensitivity analysis will be conducted for the observed sample as well as the per-protocol cohort. The EMA data collected as part of the study will be analysed using latent growth curve models, which allow for the examination of longitudinal developmental processes. These models will be estimated as structural equation models, facilitating the integration of mediators into the causal model. Significance testing of the indirect mediation effect is embedded within the model, allowing a robust analysis of how real-life social interactions, as captured by EMA, mediate the effectiveness of the group intervention.

A detailed Statistical Analysis Plan will be prepared before database lock and start of the analysis.

### Consent to participate

All prospective participants are informed about the opportunity to join the study through standardised informational materials at both study sites. If they express interest, a screening appointment will be scheduled, during which voluntary oral and written informed consent for study participation, as well as for the storage, evaluation and transfer of study-related data, will be obtained by research associates at the respective study centre. Participants will be thoroughly informed about their right to refuse participation or withdraw consent at any time, without the need to provide a reason. In the event of withdrawal of consent, participants will have the option to decide whether their data should be deleted or destroyed, or whether it can be used in anonymised form for this research project.

### Safety/harms

To date, there have only been a few studies on the adverse effects of outpatient group psychotherapy including worsening of symptoms, unpleasant memories and feelings, stress, anxiety, sadness or sleep problems.^37^ In a previous study, KCT integrated into an inpatient CBASP framework was also examined for potential negative effects through a qualitative data analysis.^27^ Of the 87 patients, 41 (47%) reported negative aspects of the group therapy, which were related to structural issues within the inpatient setting, external disturbances, deficits in group cohesion, doubts about the CBASP concept, and group size. In the present study, we have addressed most of these concerns by conducting outpatient closed groups that are not part of a CBASP setting and by maintaining a stable group size with a maximum of 10 patients.

In the present trial, adverse events (AEs) are defined as (a) exacerbation of symptoms, (b) appearance of new symptoms, (c) passive suicidal thoughts and (d) active suicidal plans or intentions. Serious adverse events (SAEs) comprise (a) death or (b) any life-threatening event that requires inpatient medical treatment. AEs and SAEs are regularly documented at all trial visits. During the weekly KCT group sessions, group therapists are encouraged to carefully notice any (S)AEs and, if necessary, interview the patient using a paper-and-pencil version of the eCRF documentation. If a patient reports an (S)AE via eCRF, the patient is going to be contacted by the study team within 24 hours to assess the (S)AE in more depth regarding the circumstances, its duration (start/end date), intensity (mild, moderate, severe), assessment of causality (treatment related, probably related, unlikely related, not related, not assessable), the actions taken, and its outcome. Additionally, the patients’ individual CBT therapist will be informed. All SAEs, particularly severe adverse treatment reactions (SATRs), have to be reported to the principal and coprincipal investigators (AG, E-LB) and will be discussed with a Data Safety Monitoring Board. Suicidality and hospitalisation in reaction to the group intervention (SATR) are defined as individual stop criteria leading to discontinuation of the trial. The whole trial will be discontinued in case the study (co)principal investigators or members of the Data Safety Monitoring Board have serious ethical concerns regarding participants’ safety.

Data monitoring will be regularly conducted by a clinical monitor from CTO to ensure patients’ safety and integrity of the clinical data in adherence to the study protocol, data quality and accuracy. The process will be independent from investigators and the sponsor.

### Ethics and dissemination

Positive ethical approval for the study and the study add-on with EMA was granted by the ethics committee of Charité Berlin (numbers: EA4/154/23, EA4/228/23) and the local ethics committees of the University of Greifswald and the Medical School of Berlin. The research outcomes will be disseminated through publication in international scientific journals and will also be shared with both the psychological and medical scientific communities through conference presentations.

### Trial status

The study officially began in April 2023. The first patient was included in July 2024. The last patient entry is expected for March 2026 and data collection aims to be completed in September 2026.

## Discussion

The transdiagnostic perspective on mental disorders has gained significant attention in clinical interventions, as demonstrated by an exponential increase in publications from 2018 (n=224) to 2023 (n=1033) (Cochrane search terms: “transdiagnostic” AND “treatment”; search date: 3 January 2024). Despite the recognised impact of social relationships on health, surprisingly few studies within this framework have focused on interpersonal functioning. Individuals who are socially isolated, are lonely or live alone are at an increased risk of premature mortality, with this risk comparable to well-established factors such as physical inactivity, obesity or substance abuse.^38^ The KCT provides techniques ideally suited for a transdiagnostic group intervention focusing on interpersonal problems. KCT has demonstrated its feasibility and effectiveness in improving interpersonal distress, even in challenging cases with treatment resistance, comorbidities and child maltreatment.^27^ We among others found that the reduction of interpersonal distress through treatment was associated with reduced depressive symptoms.^2639^,^41^ However, questions remain regarding the feasibility and efficacy of KCT in improving interpersonal problems for various diagnoses beyond persistent depressive disorder. Furthermore, it remains to be seen whether KCT will outperform the improvements achieved by individualised, state-of-the-art CBT, which is considered the gold standard in psychotherapy, despite the fact that more than half of patients do not achieve a response (eg, 42% CBT response rate in depression).^42^ Given that interpersonal problems can hinder therapeutic outcomes,^9^ it can be anticipated that an augmentation therapy focused on improving interpersonal functioning will lead to higher response rates and greater overall well-being. Unlike CBT, which addresses interpersonal functioning as one of many treatment foci, KCT delves into the individual biographical background and aims to correct interpersonal experiences through therapist and peer interactions, which is particularly significant for patients with interpersonal fears. Daily assessments of social contacts and their quality as mediators of interpersonal functioning, along with the consideration of child maltreatment as a moderator, will enhance our understanding of the impact that social interactions have on psychopathology.

### Protocol amendments

The CI has the authority to modify the study protocol if necessary, even after the study has commenced. Any significant changes affecting patient safety, the scientific basis of the study, or other critical aspects will be communicated to the relevant ethics committees. The amended protocol can only be implemented after receiving a positive opinion from the ethics committee, the signature of the amendments by the original protocol signatories, and confirmation of receipt/approval of the amendments by all investigators.

## supplementary material

10.1136/bmjopen-2024-098466 online supplemental file 1

10.1136/bmjopen-2024-098466 online supplemental file 2
