# Supplementary material for: Cognitive behavioural therapy plus Kiesler Circle Training (CBT+) versus CBT only for patients with interpersonal problems: study protocol for a randomised controlled feasibility trial
Source: BMJ Open. 2025 Feb 11;15(2):e098466. doi: 10.1136/bmjopen-2024-098466 (PMC11815471; doi:10.1136/bmjopen-2024-098466)
Supplement: online supplemental file 1 [file bmjopen-15-2-s001.docx]

**Supplementary table 1.** Exploratory outcome measures

| **Exploratory Outcomes** | | | |
| --- | --- | --- | --- |
| Impact Message Inventory, revised version ^1^ German: ^2^ | | The IMI-R will be conducted by blinded study raters. The IMI-R uses 64 items (8 items for each Kiesler Circle position) to capture covert reactions that a person evokes in a blinded-rater, so that an individual Kiesler Circle profile for each patient can be calculated. The assessment of each behavior is conducted on a 4-point scale (1 = strongly disagree; 4 = strongly agree), so that higher values mean a higher expression of the Kiesler Circle position. | |
| Beck Depression Inventory, simplified BDI-V; ^3^ German: ^4^ | | The BDI-V is a self-report questionnaire designed to assess the severity of depressive symptoms. Patients rate the severity of 20 depression symptoms on a 6-point scale (0 = never, 5 = almost always). The total score ranges from 0 to 100, with higher scores indicating a higher severity of depression. | |
| Questionnaire on panic-related Anxieties, Cognitions and Avoidance ACA; ^5^ German: ^6^ | | The ACA is a self-report questionnaire of which we assess two subdomains in our study: the Body Sensations Questionnaire (BSQ) and the Agoraphobic Cognitions Questionnaire (ACQ). The BSQ asks about anxiety regarding 17 physical symptoms and the ACQ about 14 catastrophizing thoughts that can occur during anxiety. Answers are given on a 5-point scale, with higher scores indicating higher levels of anxiety (1 = not anxious/never, 5 = extremely anxious/always). A mean value is calculated for each of the two subdomains. | |
| Short Emotional Disorder Inventory SEDI; adopted short version of MEDI by ^7^ | | The SEDI is a short form of the Multidimensional Emotional Disorder Inventory (MEDI; Rosellini & Brown, 2019), which represents a dimensional approach to classify emotional disorders. The SEDI is a self-report questionnaire comprising 15 items that are answered on a 4-point scale (1 = completely untrue or slightly true, 4 = completely true). A mean value is calculated over all items, with higher values indicating a higher level of emotional disorder traits. | |
| Patient Health Questionnaire PHQ-15; ^8 9^ and Generalized Anxiety Disorder 7: GAD-7; ^10^ | | The PHQ-9, the PHQ-15 and the GAD-7 are based on the Patient Health Questionnaire or are part of it. The PHQ-9 measures depressive symptoms using 9 items, the PHQ-15 the severity of somatic symptoms using 15 items and the GAD-7 anxiety symptoms using 7 items. The rating scales of the PHQ-9 and the GAD-7 are 4-point (0 = never, 3 = almost daily), the rating scale of the PHQ-15 is 3-point (0 = not affected, 2 = severely affected). A total score ranging from 0-24 (PHQ-9), 0-21 (GAD-7) and 0-30 (PHQ-15) is calculated for each scale. | |
| World Health Organization Quality of Life WHOQOL; ^11^ | | The WHOQoL-BREF is a self-report measure and assesses the subjective quality of life across physical, psychological, social, and environmental domains using 26 items. These domains are rated by patients on a 5-point scale and a mean score for each domain is calculated by multiplying the mean value of all items of the respective domain by four. The possible values for all domain scores therefore range from 4-20. | |
| Personality Inventory for DSM-5 Brief Form Plus PID5BF+M; ^12^ | | The PID5BF+M is a self-report questionnaire designed to assess maladaptive personality traits in alignment with the DSM-5 and ICD-11. The questionnaire comprises 36 items, which are answered on a 4-point scale (0 = completely untrue, 3 = completely true) and captures six personality trait domains (negative affectivity, detachment, antagonism/dissociality, disinhibition, anankastia, and psychoticism). A mean value is calculated for each domain, with higher scores indicating higher expression of that personality domain. | |
| Level of Personality Functioning Scale-Brief Form 2.0 LPFS-BF; ^13^ | | The LPFS-BF is a self-report questionnaire designed to assess personality functioning in alignment with the DSM-5 and ICD-11. The questionnaire comprises 12 items, which are answered on a 4-point scale (1 = completely untrue, 4 = completely true) and captures two personality functioning domains (self and interpersonal pathology). A mean value is calculated for each domain, with higher scores indicating higher expression of that personality functioning domain. | |
| Interpersonal Motives Inventory IIM; ^14^ German: ^15^ | | The IIM assesses individual motives on eight subscales: self-assurance, assertiveness, self-centeredness, reservedness, submissiveness, altruism, harmony and social acceptance. The inventory comprises a total of 64 items, rated on a 5-point scale (1 = not, 5 = very). Mean values for each of the subscales are calculated, with higher total scores indicating stronger expressions of the respective motives. | |
| Certainty About Mental States Questionnaire ^16^ | | The CAMSQ is a self-report measure assessing the perceived capacity to understand mental states of both oneself and others (mentalizing). It consists of two subscales (self-certainty and other-certainty) and 20 items, rated on a 7-point scale (1 = never, 7 = always). Mean values are calculated for both subscales, with higher scores indicating higher certainty about mental states of oneself or others. | |
| Inventory for the Assessment of Negative Effects of Psychotherapy INEP; ^17^ | | The INEP is a self-report assessment of negative changes caused by psychotherapy in the areas of intrapersonal change, relationships, friendships, family, malpractice, and stigmatization. The INEP contains 21 items, which responses are partly formulated in a 7-point bipolar format (-3 = negative change is completely true, 0 = unchanged, 3 = positive change is completely true), and partly formulated in a 4-point unipolar format (0 = completely untrue; 3 = completely true). In addition, patients' attributions regarding the cause of negative effects is assessed. For the evaluation, only negative effects directly attributed to the psychotherapeutic treatment are considered. | |
| Questionnaire for the Assessment of Side Effects and Negative Experiences in Group Therapy NUGE-24; ^18^ | | The NUGE assesses adverse experiences with group psychotherapy and side effects of the group setting by differentiating between group-related and therapist-related stress, stress from co-patients and personal overload. The NUGE comprises 24 items and responses are given on a 5-point scale (1 = strongly disagree, 5 = strongly agree), with higher scores indicating higher distress. The NUGE will be completed after each KCT group session by patients randomly assigned to the intervention group. | |
|  | |  | |

**References**

1. Kiesler D, Schmidt J. Manual for the impact message inventory-circumplex (IMI-C). *Menlo Park, CA: Mind Garden* 2006

2. Caspar F, Berger T, Fingerle H, et al. Das deutsche IMI. Das Impact Message Inventory nach Kiesler und seine Circumplexstruktur. *Psychotherapie im Dialog* 2016;17(4):1-10.

3. Beck AT, Ward CH, Mendelson M, et al. An inventory for measuring depression. *Archives of general psychiatry* 1961;4(6):561-71.

4. Schmitt M, Maes J. Vorschlag zur Vereinfachung des Beck-Depressions-Inventars (BDI): Diagnostica 2000:38–46.

5. Chambless DL, Caputo GC, Bright P, et al. Assessment of fear of fear in agoraphobics: The Body Sensations Questionnaire and the Agoraphobic Cognitions Questionnaire. *Journal of Consulting and Clinical Psychology* 1984;52(6):1090-97. doi: 10.1037/0022-006X.52.6.1090

6. Ehlers A, Margraf J, Chambless D. Fragebogen zu körperbezogenen Ängsten, Kognitionen und Vermeidung: AKV: Beltz-Test 2001.

7. Bartholdy S, Raphaela Z, Hermann A, et al. Validation of the Factor Structure and Psychometric and Clinical Properties of the Multidimensional Emotional Disorder Inventory (MEDI)-German Version. 2024 doi: <https://doi.org/10.31219/osf.io/at7p9>

8. Kroenke K, Spitzer RL, Williams JB. The PHQ‐9: validity of a brief depression severity measure. *Journal of general internal medicine* 2001;16(9):606-13.

9. Kroenke K, Spitzer RL, Williams JB. The PHQ-15: validity of a new measure for evaluating the severity of somatic symptoms. *Psychosomatic medicine* 2002;64(2):258-66.

10. Spitzer RL, Kroenke K, Williams JB, et al. A brief measure for assessing generalized anxiety disorder: the GAD-7. *Archives of internal medicine* 2006;166(10):1092-97.

11. WHOQOL Group. Development of the World Health Organization WHOQOL-BREF quality of life assessment. *Psychological medicine* 1998;28(3):551-58.

12. Bach B, Kerber A, Aluja A, et al. International Assessment of DSM-5 and ICD-11 Personality Disorder Traits: Toward a Common Nosology in DSM-5.1. *Psychopathology* 2020 doi: 10.1159/000507589

13. Spitzer C, Müller S, Kerber A, et al. Die deutsche version der level of personality functioning scale-brief form 2.0 (LPFS-BF): faktorenstruktur, konvergente validität und normwerte in der allgemeinbevölkerung. *PPmP-Psychotherapie· Psychosomatik· Medizinische Psychologie* 2021;71(07):284-93.

14. Locke KD. Circumplex scales of interpersonal values: Reliability, validity, and applicability to interpersonal problems and personality disorders. *Journal of Personality Assessment* 2000;75(2):249-67. doi: 10.1207/S15327752JPA7502_6

15. Thomas A, Locke KD, Strauß B. Das Inventar zur Erfassung interpersonaler Motive (IIM). *Diagnostica* 2012

16. Müller S, Wendt LP, Zimmermann J. Development and validation of the Certainty About Mental States Questionnaire (CAMSQ): A self-report measure of mentalizing oneself and others. *Assessment* 2023;30(3):651-74. doi: <https://doi.org/10.1177/10731911211061280>

17. Ladwig I, Rief W, Nestorius Y. Welche Risiken und Nebenwirkungen hat Psychotherapie? - Entwicklung des Inventars zur Erfassung Negativer Effekte von Psychotherapie (INEP). *Verhaltenstherapie* 2014;24:252-63.

18. Strauß B, Drobinskaya A. First experiences with the "Questionnaire for the assessment of side effects and negative experiences in group therapy" (NUGE-24). *Psychotherapie Psychosomatik Medizinische Psychologie* 2018;68:437-42.
