# Supplementary material for: Cognitive behavioural therapy plus Kiesler Circle Training (CBT+) versus CBT only for patients with interpersonal problems: study protocol for a randomised controlled feasibility trial
Source: BMJ Open. 2025 Feb 11;15(2):e098466. doi: 10.1136/bmjopen-2024-098466 (PMC11815471; doi:10.1136/bmjopen-2024-098466)
Supplement: online supplemental file 2 [file bmjopen-15-2-s002.docx]

Supplementary table 2. Trial registration Data Set.

| **Data category** | **Information** |
| --- | --- |
| Primary registry and trial identifying number | Clinical Trials: NCT06170801 |
| Date of registration in primary registry | Clinical Trials: 12.12.2023 |
| Secondary identifying numbers | German Clinical Trials Register: DRKS00032467 (25.08.2023) |
| Source(s) of monetary or material support | Charité - Universitätsmedizin Berlin |
| Primary sponsor | Charité - Universitätsmedizin Berlin Department of Psychiatry and Neurosciences  Charitéplatz 1  10117 Berlin, Germany |
| Secondary sponsor(s) | - |
| Contact for public queries | Dr. Anne Guhn  Charité – Universitätsmedizin Berlin [anne.guhn@charite.de](mailto:anne.guhn@charite.de) |
| Public title | GRoup Intervention for InterPersonal Skills -GRIPS |
| Scientific title | Efficacy and feasibility of a transdiagnostic augmentation therapy for improving interpersonal skills using the Kiesler Circle Training (KCT) |
| Countries of recruitment | Germany |
| Health condition(s) or problem(s) studied | Primary anxiety or depressive disorder according to DSM-5 with significant interpersonal problems |
| interventions | Intervention group:  Individual Cognitive Behavioral Therapy (CBT) plus  Kiesler Circle Training (1 individual session + 12 weekly group sessions of 100 minutes)  Active control group:  Individual CBT |
| Key inclusion and exclusion criteria | Age: 18-70 years, sex: all  Inclusion criteria: written informed  consent, sufficient german language skills, primary anxiety or depressive disorder, significant interpersonal distress |
|  | Exclusion criteria: Acute suicidality, active substance abuse, autism, borderline, antisocial, schizoid and schizotypic personality disorder, insufficient German language skills, any kind of additional group treatment including self-help groups |
| Study type | interventional |
| Date of first enrolment | July 2024 |
| Target sample size | 156 |
| Recruitment status | recruiting |
| Primary outcome | Change in interpersonal distress according to self-report (Inventory for Interpersonal Problems, IIP-D) |
| Key secondary outcome | Change in psychopathology regarding primary diagnoses (Hamilton Rating Scale for Anxiety, HAM-A, or Depression, HAM-D) |
| Moderator | Child maltreatment (Childhood trauma questionnaire, CTQ) |
| Mediator | Quantity and quality of social interactions |
